# Transcatheter Embolization for Giant Splenic Artery Aneurisms: Still an Open Question

**DOI:** 10.1155/2012/652469

**Published:** 2012-07-30

**Authors:** Marianna Mastroroberto, Sonia Berardi, Matteo Renzulli, Caterina Maggioli, Paolo Pianta, Antonio Daniele Pinna, Rita Golfieri, Claudia Sama

**Affiliations:** ^1^Unit of Liver Transplantation, Department of Digestive Disease and Internal Medicine, 40138 Sant'Orsola-Malpighi, Italy; ^2^Department of Emergency/Urgency, General Surgery and Transplantation, 40138 Sant'Orsola-Malpighi, Italy

## Abstract

Transcatheter embolization is the mainstay of the therapy of splenic artery aneurysms (SAAs) in patients with portal hypertension. It is indicated when the SAA diameter reaches 20 mm. Although endovascular techniques are effective and safe for the treatment of medium-sized SAAs, little is known about their applicability to large-sized SAAs. Herein, we report a case of giant SAA, which was treated with transcatheter coil embolization. The case was not considered suitable for surgery because of the presence of severe portal hypertension. The procedure was complicated by bacterial infection of the coils within the aneurismatic sac, leading to the development of hepatic failure. A liver transplant was then successfully performed despite the presence of a nonresponsive infection.

## 1. Introduction

Portal hypertension is associated with the development of splenic artery aneurysms (SAAs) [1–3], where the risk of spontaneous rupture increases with the aneurysm diameter. SAAs treatment is indicated when the aneurysm diameter reaches 20 mm. Although open surgery has been the mainstay of therapy, in recent years endovascular techniques became the first-line therapy. The mean diameter of SAAs treated with endovascular techniques is usually below 40 mm [4, 5], while the role of these techniques for bulkier SAAs is less well defined. Herein, we report a case of an unusually voluminous SAA in a patient with portal hypertension treated with transcatheter coil embolization.

## 2. Case Report

**Figure 1:**
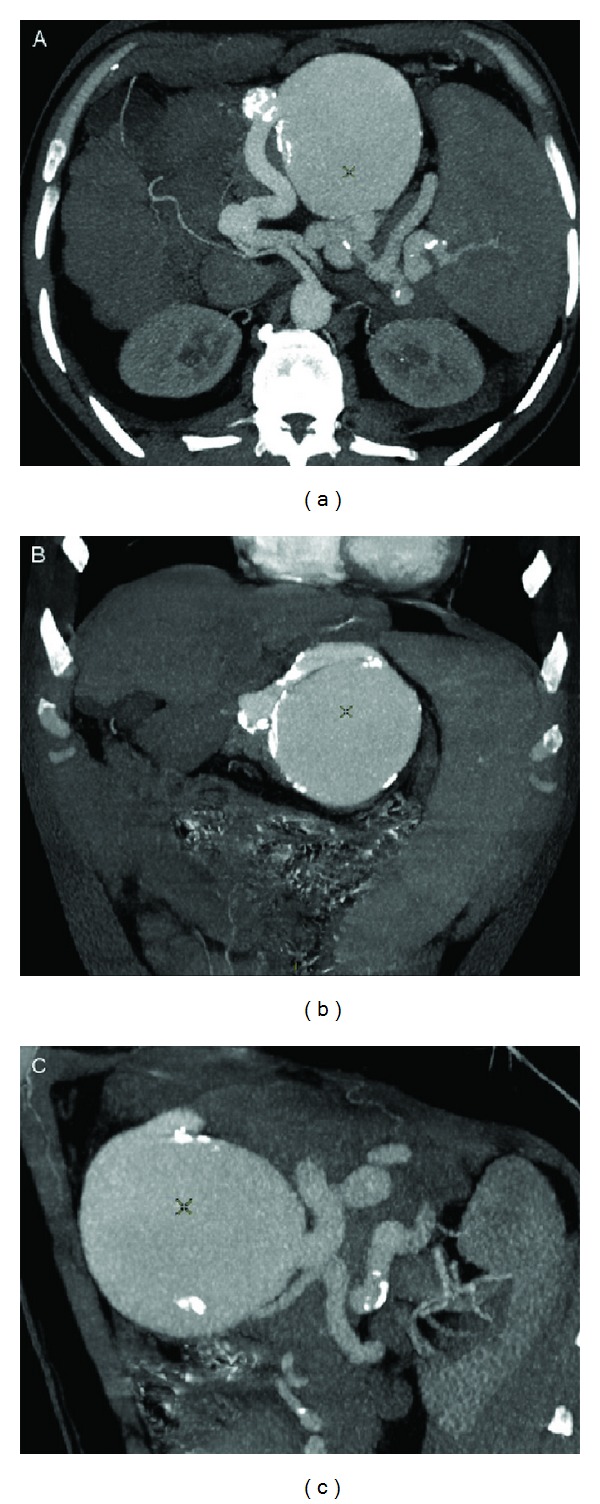
Computed tomography (CT) of the abdomen showing a tortuous and ectasic splenic artery with some widely dilated sections (max 24 mm calibre) and massive aneurysmal dilation in the middle tract (90 mm of maximum diameter) with partly calcified walls, inducing a cranial displacement of the tail and the body of the pancreas.

In August 2009, a 60-year-old man with cryptogenetic cirrhosis was referred to our centre. His history revealed splenomegaly from the age of 16, with secondary thrombocytopenia. No clinical signs of decompensation of the liver disease were present: MELD score was 13 and Child-Pugh-Turcotte score B7. Physical examination showed splenomegaly and the presence of a pulsing voluminous mass, and vascular murmur in the middle and left upper abdominal quadrants was noted. Platelet count was 26.000/microL (normal values: 150.000–380.000/microL), and small esophageal varices were documented at endoscopy. The patient underwent a computed tomography (CT) of the abdomen which showed liver cirrhosis with signs of portal hypertension, a tortuous and ectasic splenic artery with some widely dilated sections (max 24 mm caliber), and massive aneurysmal dilation in the middle tract (90 mm of maximum diameter) with partly calcified walls, inducing a cranial displacement of the tail and the body of the pancreas (Figure 1).

Because of the severe portal hypertension, the patient was not considered eligible for the surgical treatment of SAA. We therefore decided to proceed with an angiographic approach.

**Figure 2:**
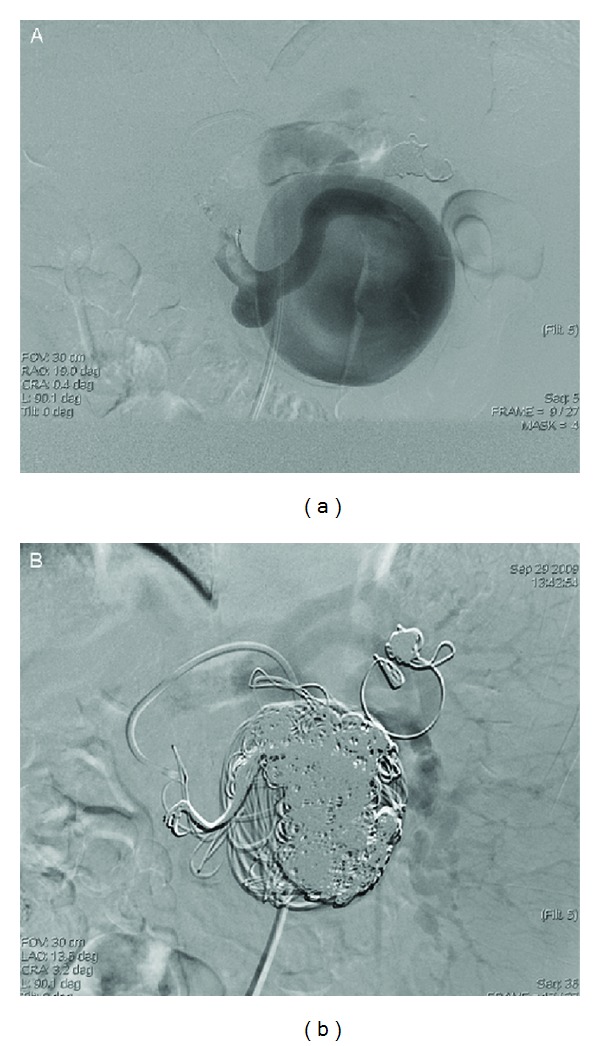
The placement of 11 guidewires and 75 coils within the aneurysmal sac.

**Figure 3:**
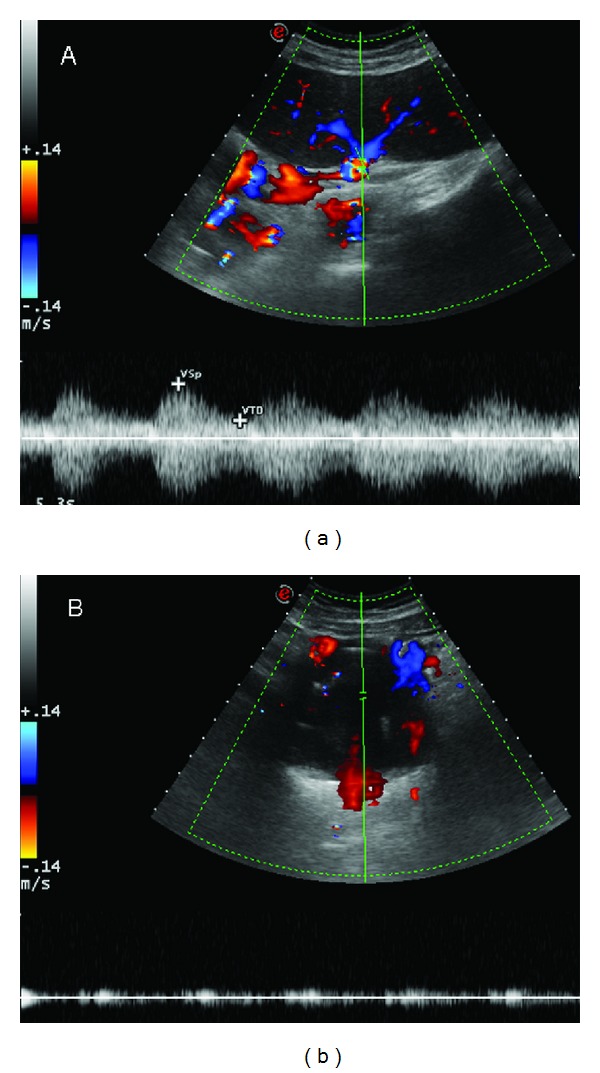
Abdominal ultrasound confirming the exclusion of the SAAs with complete preservation of distal flow to the spleen.

The angiographic study, performed via femoral artery approach, confirmed a voluminous wide-necked aneurysm located in the proximal third of the splenic artery, at 40 mm from the ostium, and revealed the presence of a second smaller SAA communicating with the former. The placement of a covered stent to straddle the aneurysms failed because of the extremely tortuous efferent splenic artery. A different approach was therefore attempted. The splenic artery blood flow was controlled by the inflation of a balloon catheter via occlusion of the proximal artery of the aneurysms to reduce the risk of aneurysm rupture and migration of the coils. This allowed the placing of 11 guidewires (“J” Curved Moveable Core, Boston Scientific, Natick, MA, USA) and 75 coils of different shape, dimensions (diameter range 10–16 mm; length range 120–300 mm) and companies (Boston Scientific, Natick, MA, USA; Cook, Bloomington, IN, USA; Balt, Montmorency, France) within the aneurysmal sac. The exclusion of the SAAs with complete preservation of distal flow to the spleen was thus obtained (Figure 2), as confirmed by abdominal ultrasound (Figure 3).

A broad-spectrum antibiotic was given intravenously before, during and after the procedure. The patient was discharged from the hospital 7 days later in a good clinical condition.

**Figure 4:**
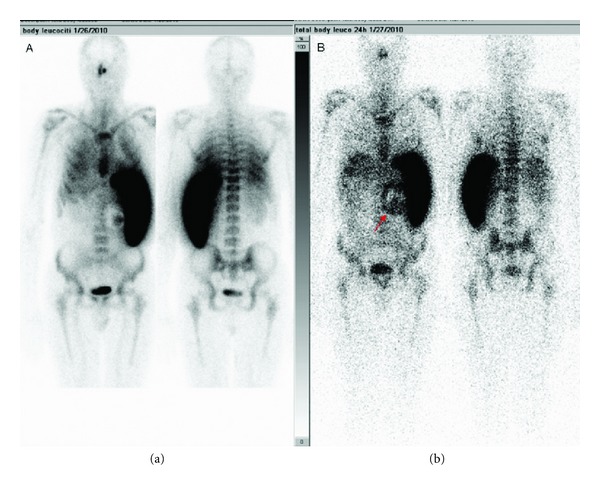
Tc-99m labelled white cells scintigraphy showing a progressive accumulation of circular leucocytes (90 × 90 mm) with sonolucent halo surrounding the aneurysm wall.

One month after the embolization, the patient developed high-moderate grade fever (>38°C). Although large-spectrum antibiotic therapy was rapidly started, the fever worsened and the patient was again hospitalized in our unit. Abdominal ultrasound and CT scans excluded strokes/spleen abscesses, while a Tc-99m labelled white cells scintigraphy showed a progressive accumulation of circular leucocytes (90 × 90 mm) with a sonolucent halo surrounding the aneurysm wall (Figure 4). A blood culture was positive for Propionibacterium bacterium acnes, sensitive only to aminoglycosides, vancomycin, and clindamycin. Diagnosis of suprainfection of vascular devices was therefore suspected.

Despite adequate antibiotic therapy, the clinical conditions worsened and liver failure developed (MELD 22, Child-Pugh-Turcotte C11). Because of the low pathogenicity and virulence of the causative bacteria, and the fact that infection was confined within the aneurysmatic sac, we decided to place the patient on the waiting list for a liver transplantation.

The transplantation was performed in March 2010. During the operation, an en bloc removal of 2/3 of the distal stomach, spleen, tail and body of the pancreas and aneurysmatic bags was required because the pancreas and the transverse colon were tenaciously adherent to aneurysms. Furthermore, a partial resection of the transverse colon followed by a laterolateral anastomosis was performed due to the presence of extensive intestinal injury.

The postoperative period was complicated by the development of a pancreatic fistula, which was drained through the laparotomic wound and in which was resolved gradually over the following weeks, and by a partial thrombosis of the superior mesenteric vein, which was resolved through an oral anticoagulant therapy.

The patients was discharged 27 days after liver transplantation and, after two years, he is asymptomatic and in good clinical condition.

## 3. Discussion

Splenic artery aneurysms are the most common visceral artery aneurysms, with a reported prevalence of 0.8% at arteriography and 0.04–0.10% at autopsy. The prevalence of a splenic artery aneurysm is substantially increased in patients with portal hypertension, and it is estimated to be at 7–20% in patients with cirrhosis [1]. Spontaneous rupture risk increases with aneurysm diameter, and reasonable preventive measures are indicated when the aneurysm diameter reaches 20 mm [1–5].

Intravascular therapy, including stent implantation and embolization, is today considered the treatment of choice for SAAs. Stent implantation the straddling of the aneurysm, preserving the artery splenic blood flow [4]. Various embolization techniques exist. may The* isolation technique *consists of embolizing both the inflow and the outflow arteries of the aneurysm. To avoid total splenic infarction, this strategy may be used only in presence of collateral arteries, which ensure the splenic flow and cannot be applied to aneurysms located at the origin of the splenic artery. Alternatively, in the *packing technique *a framework with interlocking detachable coils is established, and the fibered coils or microcoils are packed in the aneurysm itself until there is no blood flow. A balloon catheter is used to control splenic arterial blood flow during the procedure. Finally, a combination of the isolation and packing techniques is used if there are intrasplenic branches that originate from the aneurysm, in order to prevent recanalization due to retrograde backflow from the vessels of the splenic side.

The primary technical success rate of transcatheter embolization is 88%, reaching 100% after a second treatment [4]. The most frequent complication is the postembolization syndrome, such as transient fever and pain, resolvable in little time with symptomatic treatment in most cases [4, 6]. Less frequent possible complications include transient elevation of pancreatic enzymes, splenic infarction, infection, abscess, and, rarely, the rupture of a pseudoaneurysm [1, 6]. Nevertheless, the majority of the data in the literature concerns the treatment of SAAs of a diameter of less than 40 mm, while the optimal treatment of giant SAAs is not well established. Giant SAAs are a rare entity, and only a few cases treated with transcatheter embolization are reported in literature, all with positive result [7–10].

We have described an unusually voluminous SAA occurring in a patient with portal hypertension. The clinical history, the large size of the aneurysm, and the relatively preserved liver function led us to the hypothesis that the underlying liver disease was likely to be a congenital hepatic fibrosis, and that the aneurysm had developed very slowly over time. The aneurysm size dictated the need for intervention. In our case, the severe thrombocytopenia and portal hypertension meant that the surgical approach would involve very high risks, and therefore an angiographic treatment was chosen. The initially planned placement of a covered stent failed because of the extremely tortuous efferent splenic artery. The isolation technique was not considered because of the absence of adequate collaterals.

We therefore chose the packing technique with the use of a balloon catheter to control splenic arterial blood flow during the procedure. We succeeded in eliminating the SAAs with complete preservation of distal flow to the spleen.

However, the procedure was complicated a few weeks later by a bacterial contamination of the devices through *Propionibacterium acnes*. This complication, uncommon in the literature data, was probably favoured in our patient by the high number of coils utilized, which also made the penetration of effective antibiotics at the outbreak of the infection extremely difficult. Because the infection could not be cured by other means, and the subsequent development of hepatic failure, the only possibility for our patient was liver transplantation. The adhesions occurring as a result of an infection of the aneurysmal sac required a visceral extended resection, but this did not compromise the success of transplantation.

In conclusion, even though therapeutic comparison choices appear well-codified today for medium-sized SAAs, further studies are needed to formulate an algorithm for the optimal management of giant SAAs, a rare but potentially life-threatening entity. In the case of transcatheter embolization, the risk of a microbial contamination of the numerous devices utilized, with the subsequent difficult penetration of antibiotics at the outbreak of the infection, should be taken in account.
